# Effect of mildly elevated thyroid-stimulating hormone during the first trimester on adverse pregnancy outcomes

**DOI:** 10.1186/s12902-018-0294-7

**Published:** 2018-09-12

**Authors:** Ping Li, Shuo Lin, Ling Li, Jinhui Cui, Shuisheng Zhou, Jianhui Fan

**Affiliations:** 1 Department of Obstetrics and Gynecology, The Third Affiliated Hospital of Sun Yat-Sen University, No. 600, Tianhe road, Guangzhou, 510630 China; 2 Department of Endocrinology, the Third Affiliated Hospital of Sun Yat-Sen University, Guangzhou, China

**Keywords:** Thyroid-stimulating hormone, First trimester, Adverse pregnancy outcomes

## Abstract

**Background:**

To investigate the effect of a mildly elevated thyroid-stimulating hormone (TSH) concentration between 2.5 and 4.0 mIU/L during the first trimester on pregnancy outcomes in thyroid peroxydase antibody (TPOAb)-negative pregnant women.

**Methods:**

A total of 1858 pregnant women who were TPOAb-negative before 13^+ 6^ gestational weeks, received regular prenatal services, and delivered in the third affiliated hospital of Sun Yat-Sen University were recruited from June 2016 to June 2017. Measurements of thyroid function (TSH, free T4 [FT4] and TPOAb) and adverse pregnancy outcomes were assessed and recorded.

**Results:**

Among the 1858 study participants, the 97.5th percentile for TSH was 3.76 mIU/L, and 142 women (7.6%) had mildly elevated TSH levels between 2.5 and 4.0 mIU/L. No differences in the incidence of adverse pregnancy outcomes were observed between patients with a mildly elevated TSH level and those with a normal TSH level (< 2.5 mIU/L).

**Conclusion:**

A mildly elevated TSH concentration (2.5–4.0 mIU/L) during the first trimester of pregnancy in TPOAb-negative women was not associated with adverse pregnancy outcomes in our study population. Accordingly, it may be possible to raise the upper limit of the healthy TSH reference range for pregnant women.

## Background

Thyroid diseases are common endocrine disorders among women of reproductive age [1], and the serum concentration of thyroid-stimulating hormone (TSH) is the most commonly used index for evaluating thyroid function during pregnancy. However, the normal range for serum TSH concentrations during pregnancy is not the same as that for nonpregnant women [2]. The increased serum concentration of human chorionic gonadotropin (HCG) along with the synthesis of thyroxine-binding globulin (TBG) may lead to the alteration of maternal thyroid hormone levels during early pregnancy [3], with the maternal serum TSH level being generally lower in the first trimester than in non-pregnancy and then increasing gradually.

The American Thyroid Association (ATA) guideline (2011) recommends upper limits for healthy serum TSH concentrations of 2.5 mIU/L in the first trimester and 3.0 mIU/L in the second and third trimesters [4]. Based on these diagnostic criteria, subclinical hypothyroidism (SCH), which defined as an elevated TSH concentration with a normal serum FT4 concentration, is estimated to affect up to 15% of pregnant women in the United States and 28% of pregnant women in China [5, 6]. However, many studies have demonstrated that SCH may be over-diagnosed in a large proportion of pregnant when the universal thresholds of 2.5 and 3.0 mIU/L TSH are used [6–8]. Such diagnoses may worry patients and thus add to their psychological stress. A significantly elevated TSH concentration is indeed associated with adverse maternal and neonatal outcomes, including gestational diabetes mellitus (GDM), preeclampsia, placental abruption, and preterm delivery [9–11]. However, whether a mildly elevated TSH concentration increases adverse pregnancy outcomes, especially in thyroid peroxidase antibody (TPOAb)-negative pregnant women, has been debated in recent years [12–14]. Accordingly, a more liberal upper limit for the healthy TSH range in healthy pregnant women has been proposed [15], and the 2017 ATA guideline was updated with an upper reference limit of 4.0 mIU/L TSH during pregnancy [16].

In our present study, we analyzed the normal range for TSH concentration, based on the 2.5th and 97.5th percentile values, during the first trimester in healthy pregnant women and whether a mildly elevated TSH concentration between 2.5 and 4.0 mIU/L during the first trimester increased the risk of adverse pregnancy outcomes in TPOAb-negative pregnant women.

## Methods

### Study participants

Pregnant women who had a prenatal visit before 13^+ 6^ gestational weeks were invited to undergo thyroid screening in the third affiliated hospital of Sun Yat-Sen University. A total of 1858 pregnant women who received regular prenatal services and delivered in this hospital were enrolled from June 2016 to June 2017. The exclusion criteria included the following: personal or family history of thyroid disease, multiple pregnancy, assisted reproduction, TPOAb positivity, medical history of any other chronic disease or use of any medication that may influence thyroid function. All women provided written informed consent for both participation in the study and use of their health records from follow-up. The study was approved by the Human Research Ethics Committee of the third affiliated hospital of Sun Yat-Sen University.

### Data collection

Serum samples were obtained in the morning after an 8-h fast from all study participants during the first trimester. Measures of thyroid functions, including TSH, free thyroxine (FT4) and TPOAb, were examined at the clinical analysis laboratory using an automated two-step chemiluminescent immunoassay on an ARCHITECT analyzer (Abbott Diagnostics). The reference range for TSH was defined as the 2.5th and 97.5th percentiles in TPOAb-negative women. The reference range for TPOAb (0–60 IU/ml) was provided by the assay manufacturer. Women with a TSH concentration ≥ 4.0 mIU/L were treated with levothyroxine during pregnancy, while those with a TSH concentration between 2.5 and 4.0 mIU/L were not treated with levothyroxine.

Patients’ baseline characteristics at the time of the thyroid function testing, including age and pregestational body mass index (BMI, BMI = weight (kg)/height (m^2^)) were recorded. Obstetric and neonatal outcomes were assessed and documented, including gestational age at delivery, birth method, premature delivery (a live birth before 37 weeks of gestation), GDM (one or more plasma venous glucose values ≥5.1 mmol/L at 0 h, ≥10.0 mmol/L at 1 h or ≥ 8.5 mmol/L at 2 h after a 2-h 75-g oral glucose tolerance test), gestational hypertension (blood pressure > 140/90 mmHg on at least two occasions more than 6 h apart without evidence of chronic hypertension or significant proteinuria), preeclampsia (criteria for gestational hypertension plus significant proteinuria), placenta previa (placenta completely or partially covering the internal cervical os at the time of delivery), placental abruption (premature separation of a normally implanted placenta), premature rupture of membranes (PROM, membrane rupture prior to the onset of labor), intrauterine growth restriction (IUGR, a fetal weight < 10th percentile for gestational age), postpartum hemorrhage (PPH, postpartum hemorrhage volume > 500 ml for natural birth or > 1000 ml for cesarean section), low birth weight (LBW, a live birth weight ≤ 2.5 kg), small for gestational age (SGA, <10th percentile of weight in grams for gestational age by gender), large for gestational age (LGA, >90th percentile of weight in grams for gestational age by gender), and low Apgar score (≤7 at 1 or 5 min).

Women who experienced spontaneous abortion (abortion before 20 weeks of gestation) had no obstetric and neonatal data, and the incidence of spontaneous abortion was analyzed separately.

### Statistical analysis

Data are presented as mean (standard deviation [SD]) for normally distributed data, as median (interquartile range) for non-normally distributed data, and as frequency (percentage) for categorical variables. Mann-Whitney test or Chi-square test was used to test for differences in variables between groups. *P* < 0.05 was considered statistically significant. SPSS19.0 software (SPSS, Inc., Chicago, IL) was used for all statistical analyses.

## Results

**Fig. 1:**
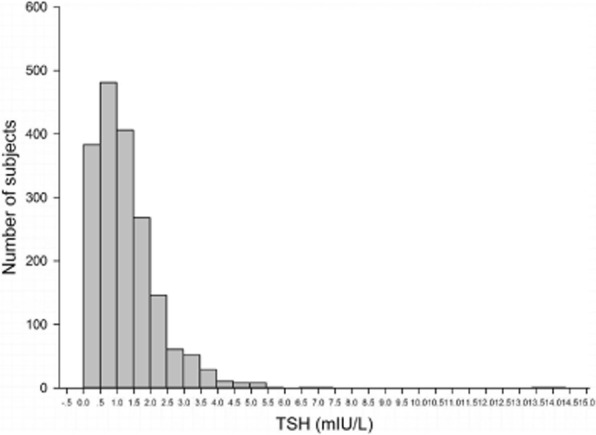
Distribution of thyroid-stimulating hormone concentrations during the first trimester among the entire study cohort (*N* = 1858)

The age, pregestational BMI, and results of thyroid function tests during the first trimester of pregnancy for all study participants are presented in Table 1. Of the 1858 pregnant women included in the study cohort, the median age was 30 (27–34) years and the median pregestational BMI was 20.03 (18.80–21.51 kg/m^2^). There were 153 women (8.2%) with TSH < 0.1 mIU/L, 1532 women (82.5%) with 0.1 mIU/L ≤ TSH < 2.5 mIU/L, 142 women (7.6%) with 2.5 mIU/L ≤ TSH < 4.0 mIU/L, and 31 women (1.7%) with TSH ≥4.0 mIU/L. The median FT4 concentration was 16.52 pmol/L (14.46–18.73 pmol/L). According to the distribution of first-trimester TSH values in the study cohort (Fig. 1), the mean, median, 2.5th and 97.5th percentile values were 1.25, 1.07, 0.01 and 3.76 mIU/L, respectively. Therefore, the normal range based on the 2.5th and 97.5th percentiles was 0.01–3.75 mIU/L.

**Table 1:** Characteristics of the study participants

Characteristic	All women (*N* = 1858)^a^
Maternal age (years)	30 (27–34)
Pregestational BMI (kg/m^2^)	20.03 (18.80–21.51)
TSH (mIU/L), *n* (%)	1.07 (0.58–1.68)
< 0.1	153 (8.2)
≥0.1 and < 2.5	1532 (82.5)
≥2.5 and < 4.0	142 (7.6)
≥4.0	31 (1.7)
FT4 (pmol/L)	16.52 (14.46–18.73)

^a^Median (interquartile range) or *n* (%)

*BMI* body mass index, *TSH* thyroid-stimulating hormone, *FT4* free thyroxine 4

The demographic and clinical characteristics of pregnant women and their newborns were compared between women with a first-trimester TSH concentration less than 2.5 mIU/L (normal group) and women with a mildly elevated TSH (eTSH) concentration of 2.5–4.0 mIU/L (eTSH group; Table 2). Maternal age, gestational age at delivery, postpartum hemorrhage volume, neonatal birth weight, and blood glucose levels at 0 and 2 h during the OGTT were similar between the two groups (*P* > 0.05; Table 2). Compared with the normal group, the eTSH group had a significantly lower pregestational BMI (19.32 ± 1.91 vs 20.03 [18.83–21.68], *P* = 0.01) and mean FT4 level (15.82 ± 2.74 vs 16.46 ± 3.11, *P* = 0.02). As excepted, the median TSH level in the normal group was significantly lower than that in the eTSH group (1.06 [0.67–1.54] vs 3.06 [2.76–3.40], *P* < 0.001).

**Table 2:** Characteristics of the study participants divided by first-trimester TSH concentration^a^

Characteristics	Normal (*n* = 1532)	eTSH (2.5–4 mIU/L) (*n* = 142)	*P*
Maternal age (years)	30 (27–34)	29 (28–32)	0.35
Pregestational BMI (kg/m^2^)	20.03 (18.83–21.68)	19.32 (±1.91)	0.01
Gestational age at delivery (weeks)	39.29 (38.57–40.00)	39.14 (38.57–40.00)	0.83
Postpartum hemorrhage volume (ml)	305 (255–380)	317 (260–380)	0.41
Neonatal birth weight (kg)	3.20 (2.95–3.50)	3.27 (±0.41)	0.05
TSH (mIU/L)	1.06 (0.67–1.54)	3.06 (2.76–3.40)	< 0.001
FT4 (pmol/L)	16.46 (±3.11)	15.82 (±2.74)	0.02
Blood glucose on 75-g OGTT (mmol/L)			
0 h	4.12 (3.94–4.36)	4.13 (3.92–4.31)	0.42
1 h	7.30 (6.16–8.46)	6.64 (5.70–7.86)	< 0.001
2 h	6.45 (5.64–7.36)	6.31 (5.58–7.01)	0.12

^a^Values are mean (SD) or median (interquartile range)

*TSH* thyroid-stimulating hormone, *eTSH* elevated thyroid-stimulating hormone, *BMI* body mass index, *FT4* free thyroxine 4, *OGTT* oral glucose tolerance test

Maternal and neonatal outcomes were also compared between the normal and eTSH groups (Table 3). No differences were observed between the two groups in adverse pregnancy outcomes, including the incidence of IUGR, GDM, gestational hypertension/preeclampsia, PROM, placental abruption, placenta previa, premature delivery, PPH, assisted vaginal delivery/cesarean section, LBW, SGA, LGA or low Apgar score (all *P* > 0.05), indicating that a mildly elevated TSH concentration during the first trimester was not associated with an increased risk of any of these pregnancy complications.

**Table 3:** Adverse pregnancy outcomes according to maternal TSH concentration in the first trimester

Pregnancy outcomes	Normal (*n* = 1532), *n* (%)	eTSH (2.5–4 mIU/L) (*n* = 142), n (%)	χ^2^	*P*
Mother				
IUGR	12 (0.8)	1 (0.7)	0.00	1.00
GDM	163 (10.6)	12 (8.5)	0.67	0.42
Gestational hypertension/preeclampsia	25 (1.6)	1 (0.7)	0.25	0.62
PROM	394 (25.7)	30 (21.1)	1.45	0.23
Placental abruption	14 (0.9)	1 (0.7)	0.00	1.00
Placenta previa	15 (1.0)	0 (0.0)	0.52	0.47
Premature delivery (< 37 weeks)	60 (3.9)	3 (2.1)	0.72	0.40
PPH	50 (3.3)	5 (3.5)	0.03	0.87
Delivery method (assisted vaginal delivery or cesarean section)	95 (25.9)	7 (23.3)	0.10	0.76
Newborn				
LBW	38 (2.5)	4 (2.8)	0.00	1.00
SGA	36 (2.3)	3 (2.1)	0.00	1.00
LGA	236 (15.4)	21 (14.8)	0.04	0.84
Apgar score ≤ 7 at 1 or 5 min	27 (1.8)	0 (0.0)	1.55	0.21

*TSH* thyroid-stimulating hormone, *eTSH* elevated thyroid-stimulating hormone, *IUGR* intrauterine growth restriction, *GDM* gestational diabetes mellitus, *PROM* premature rupture of membranes, *PPH* postpartum hemorrhage, *LBW* low birth weight, *SGA* small for gestational age, *LGA* large for gestational age

To evaluate the potential association of a mildly elevated TSH concentration and the incidence of spontaneous abortion, we separately analyzed the data collected in our hospital from June 2016 to June 2017. Ninety-four women experienced spontaneous abortion (< 20 weeks gestation), and among them, data for thyroid function measures were not available. Among the remaining 69 women, the median TSH concentration was 1.06 mIU/L (0.58–1.66 mIU/L). Of the 1927 study participants with data for thyroid function measures, the incidence rates of spontaneous abortion were 2.5% (*n* = 4) in those with TSH < 0.1 mIU/L, 3.6% (*n* = 58) in those with 0.1 mIU/L ≤ TSH < 2.5 mIU/L, 3.4% (*n* = 5) in those with 2.5 mIU/L ≤ TSH < 4.0 mIU/L, and 9.5% (*n* = 2) in those with TSH ≥4.0 mIU/L (χ^2^ = 2.66, *P* = 0.45). No statistically significant differences were observed in the prevalence of spontaneous abortion according to these categories of TSH concentration (all *P* > 0.05). These results indicated that a mildly elevated TSH concentration (2.5–4.0 mIU/L) during the first trimester was not associated with an increased risk of spontaneous abortion.

## Discussion

In the present study, the upper range for normal TSH in TPOAb-negative pregnant women during the first trimester was found to be 3.76 mIU/L, which is much higher than the 2.5 mIU/L cutoff value recommended by the 2011 ATA guideline and more consistent with the 4.0 mIU/L cutoff value now recommended by the 2017 ATA guideline. Compared with TSH < 2.5 mIU/L, a mildly elevated TSH (2.5–4.0 mIU/L) during the first trimester in TPOAb-negative women was not found to increase the frequency of adverse pregnancy outcomes. Thus, our results suggest that a normal TSH cutoff value of 2.5 mIU/L may not be suitable for Chinese pregnant women.

After the publication of the 2011 ATA guideline, researchers reported that the use of a fixed upper limit for TSH of 2.5 mIU/L during the first trimester may have led to over-diagnosis of SCH among pregnant women [17]. Data from several cohorts of pregnant women without pre-existing thyroid diseases showed that the upper first trimester TSH limit was higher than 2.5 mIU/L [6, 18, 19], which meant that a large number of women had been inappropriately diagnosed with SCH according to the 2011 ATA criteria. Higher upper limits for normal TSH during the first trimester have been reported by several studies, including 4.87 mIU/L by Li et al. [6], 4.38 mIU/L in Chen et al. [20], 4.28 mIU/L by Goldman et al. [21] and 3.76 mIU/L in our study. However, variation in TSH levels among different studies may occur due to differences in research populations, such as different ethnicities, as well as analysis methods. The optimal threshold values for TSH concentration in different populations require further study.

Whether a mildly elevated TSH concentration is associated with adverse pregnancy outcomes has remained controversial. Some studies have reported that SCH correlates with a variety of obstetric complications, including IUGR, placental abruption and GDM. In a meta-analysis of 18 studies, Maraka et al. showed that pregnant women with SCH had higher risk of pregnancy loss, placental abruption, PROM, and neonatal death compared with euthyroid pregnant women [22]. However, in their meta-analysis, the screening time for thyroid function was inconsistent among the included studies, and patients were not stratified according to TPOAb status. Still, a study by Negro et al. found that first-trimester TSH levels between 2.5 and 5.0 mIU/L, which included higher concentrations than our range [2.5–4.0 mIU/L]), in thyroid antibody-negative women were significantly associated with spontaneous abortion [23]. Tudela et al. found that women with SCH had a significantly higher risk of GDM than euthyroid women after adjustment for maternal age, race and weight [10]; however, TPOAb status was not considered in their study. In contrast, we did not include TPOAb-positive women in our study, and our results showed that a TSH concentration between 2.5 and 4.0 mIU/L during the first trimester was not related to the incidence of any of the recorded adverse pregnancy outcomes, including IUGR, GDM, gestational hypertension/preeclampsia, PROM, placental abruption, placenta previa, premature delivery, PPH, assisted vaginal delivery/cesarean section, LBW, SGA, LGA, low Apgar score, and spontaneous abortion. Li et al. also showed that when the maternal TSH concentration was within the pregnancy-specific reference range, there was no differences in mental and psychomotor development among infants, even though the TSH concentration was higher than 2.5 mIU/L [6]. According to our study and that of Hirsh et al., a TSH value well above 2.5 mIU/L did not correlate with the observed pregnancy outcomes [24]. Moreover, in a large meta-analysis of 15 studies, Sheehan et al. reported that an elevated TSH concentration during the first trimester was not associated with an increased risk for preterm delivery [25]. In addition, a recent study by Torie et al. showed that TSH ≥2.5 mIU/L during the first trimester was not associated with an increased risk of preterm delivery, GDM, or preeclampsia [26]. Together, these results suggest that the a mildly elevated TSH concentration during the first trimester is likely not associated with adverse pregnancy outcomes.

Our study adds further evidence that a cutoff of 2.5 mIU/L for TSH during the first trimester of pregnancy might not be appropriated for diagnosing SCH in Chinese pregnant women. However, there are some limitations in our research. First, this was a single-center study. Also, we did not perform thyroid ultrasonography or detect thyroglobulin antibody in the study population. These limitations may reduce the general applicability of our findings. Secondly, the numbers of cases with adverse pregnancy outcomes were few, and thus, differences between groups may not have been detected. Finally, we did not follow-up on the neurocognitive development of the infants. Thus, further research is needed to determine the optimal TSH cutoff value for diagnosing SCH.

## Conclusion

A mildly elevated TSH concentration (2.5–4.0 mIU/L) during the first trimester in TPOAb-negative women was not associated with adverse pregnancy outcomes in our study population. Therefore, the results of the present study indicate that a more liberal upper limit for the healthy TSH reference range during the first trimester of pregnancy could be recommended in healthy pregnant women without thyroid diseases.

## Abbreviations

ATA: The American Thyroid Association
BMI: Body mass index
FT4: Free thyroxine 4
GDM: Gestational diabetes mellitus
HCG: Human chorionic gonadotropin
IUGR: Intrauterine growth restriction
LBW: Low birth weight
LGA: Large for gestational age
PPH: Postpartum hemorrhage
PROM: Premature rupture of membranes
SCH: Subclinical hypothyroidism
SGA: Small for gestational age
TBG: Thyroxine-binding globulin
TPOAb: Thyroid peroxidase antibody
TSH: Thyroid-stimulating hormone
